# Distribution characteristics and influencing factors of three-dimensional lens parameters in patients with age-related cataracts

**DOI:** 10.1186/s12886-026-05062-7

**Published:** 2026-06-29

**Authors:** Haifeng Jiang, Biyue Tu, Yanxia Tong, Zhanpeng Zhu, Yong Wang

**Affiliations:** 1 Department of Ophthalmology, Renmin Hospital of Wuhan University, Wuhan University, Wuhan, Hubei Province China; 2 Aier Eye Hospital of Wuhan University (Wuhan Aier Eye Hospital), Wuhan, Hubei Province 430060 China

**Keywords:** Cataract, Lens, Three-dimensional, OCT

## Abstract

**Background:**

To characterize the three-dimensional lens parameters in age-related cataract patients and examine their associations with age, sex, and ocular biometrics.

**Methods:**

In this retrospective cohort study, we consecutively enrolled patients aged ≥ 40 years who underwent cataract surgery at the Aier Eye Hospital of Wuhan University (Wuhan Aier Eye Hospital) between January 2023 and December 2024. Three-dimensional lens parameters were acquired using swept-source anterior segment optical coherence tomography (SS-AS-OCT), including: anterior lens surface curvature radius (RAL), lens posterior surface curvature radius (RPL), lens thickness (LT), anterior lens thickness (LT_a_), lens diameter (LD), and lens volume (LV). Correlations with age, sex, and ocular biometrics were analyzed.

**Results:**

The study included 356 patients (140 males and 216 females) with a mean ± SD age of 66.48 ± 9.60 years. No significant sex-related differences were found in any lens parameters (all *p* > 0.05). RAL demonstrated a significant negative linear correlation with age (*r* = -0.142, *p* = 0.007), decreasing at a rate of 0.019 mm/year. In contrast, LT, LT_a_, and LV showed positive linear correlations with age, increasing at rates of 0.013 mm/year, 0.007 mm/year, and 1.052 mm³/year, respectively (all *p* < 0.05). Age stratification showed LT increased faster in ≤ 60 years (0.022 mm/year vs. 0.010 mm/year). RAL was negatively correlated with LT and LT_a_, but positively with RPL and LD. RPL, LT, LT_a_, LD, and LV were positively intercorrelated. Ocular biometrics showed that RAL correlated positively with ACD, AL, and WTW but negatively with K_m_. RPL positively correlated only with AL. LT, LT_a_, LD, and LV exhibited negative correlations with ACD, while LD and LV positively correlated with AL (all *p* < 0.05).

**Conclusions:**

In cataract patients aged ≥ 40 years, the anterior lens surface convexity, LT, and LV increased significantly with age, with more rapid progression rates before age 60 than thereafter. Multivariable analysis revealed that smaller ACD was associated with more pronounced anterior lens surface convexity and greater lens thickness. LT, LT_a_, and RAL showed nonlinear relationships with AL, where RAL was maximal and LT was thinnest at approximately 25 mm AL.

**Supplementary Information:**

The online version contains supplementary material available at https://doi.org/10.1186/s12886-026-05062-7.

## Introduction

The anatomical configuration of the lens is intrinsically associated with lens-related pathologies, including cataract and presbyopia [1]. Furthermore, by modifying anterior chamber volume and anterior chamber angle structure, the lens plays a pivotal role in angle-closure glaucoma and other intraocular pressure-related disorders [2]. In cataract management, accurate quantification of lens morphometric parameters critically informs surgical planning and facilitates personalized intraocular lens (IOL) selection [3]. Studies have demonstrated that spheroid-shaped lenses are primarily characterized by an increase in the curvature of the anterior and posterior lens surfaces and a reduction in the equatorial radius. These morphological features are often associated with zonular laxity or lens subluxation, necessitating specialized surgical techniques such as capsular tension ring placement [4]. Notably, larger dimensional parameters (e.g., capsular bag diameter and volume) substantially affect postoperative IOL stability and markedly elevate rotation risks for toric IOLs [5]. 

Furthermore, lens morphometric parameters significantly influence the calculation of IOL power. Modern IOL formulas (e.g., Barrett Universal II and EVO) incorporate five key ocular parameters, including lens thickness, which has significantly improved predictive accuracy [6]. However, current clinical studies predominantly focus on two-dimensional parameters such as axial lens thickness, while largely neglecting three-dimensional characteristics. This study aimed to establish a comprehensive three-dimensional lens parameter database for age-related cataract patients using swept-source anterior segment optical coherence tomography (SS-AS-OCT; CASIA 2, Tomey, Japan). We analyzed the distribution of key parameters including lens thickness, volume, and anterior/posterior surface curvature radii, investigated their correlations with age, sex, and other ocular biometric parameters, and provided a reference for precise cataract diagnosis and treatment.

## Methods

### Patients

In this retrospective cohort study, we reviewed medical records of patients who underwent cataract surgery at Aier Eye Hospital of Wuhan University between January 2023 and December 2024. To avoid data duplication, only one eye per patient was included (the right eye was selected when both eyes were eligible).

Inclusion criteria were: (1) age ≥ 40 years; and (2) diagnosis of age-related cataract. Exclusion criteria included: (1) history of ocular surgery (except uncomplicated phacoemulsification); (2) lens dislocation/subluxation; and (3) dense cataracts precluding accurate biometric measurements.

The study obtained approval from the Ethics Committee of Aier Eye Hospital of Wuhan University, and all study procedures complied with the Declaration of Helsinki.

### Lens parameter measurement

Measurement of lenticular parameters was performed using the anterior segment optical coherence tomography (AS-OCT; CASIA 2, Tomey, Japan), which acquired 16 tomographic images of the lens at different cross-sectional positions spanning 0°–360°. All examinations were performed independently by two experienced operators.

The device utilizes swept-source Fourier-domain optical coherence tomography (OCT) technology with a 1310 nm measurement wavelength and a scanning speed of 50,000 A-scans/second. It rapidly captures three-dimensional images from the corneal surface to a depth of approximately 13 mm at the posterior lens surface within 0.3 s, covering an anterior segment area of 16 × 13 mm (width × depth). During the procedure, patients seated with their lateral canthi aligned with the device’s eye-level marker. Patients were instructed to fixate on the indicator light while the operator aligned the crosshair on the device screen with the corneal apex to capture anterior segment scan images. Examination results were retained only when three consecutive measurements displayed “OK” quality indicators. Anterior segment parameters were automatically measured and calibrated using built-in algorithms. Measurement procedures were as follows:

#### Parameters automatically tracked and quantified by the CASIA 2 device (3D mode)

**Fig. 1:**
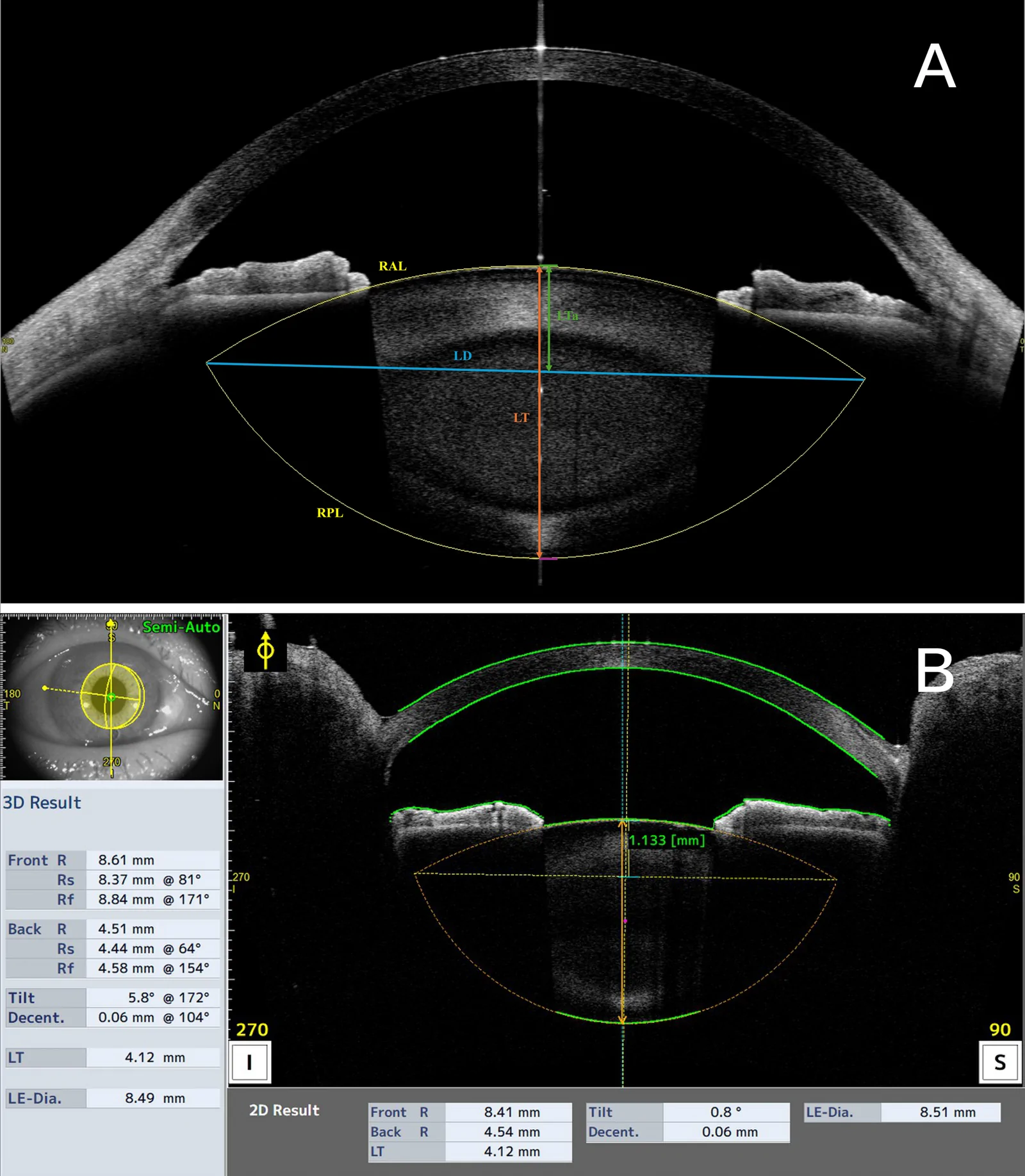
Schematic of lens-related parameter measurement and acquisition. **A** Shows the structural schematic of lens parameters in the 0°–180° two−dimensional section. The yellow arc (anterior lens surface curvature radius, RAL) indicates the anterior lens surface radius of curvature. The yellow arc (posterior lens surface curvature radius, RPL) indicates the posterior lens surface radius of curvature. The blue line (lens diameter, LD) indicates the equatorial diameter of the lens. The orange line (lens thickness, LT) indicates lens thickness. The green line (anterior lens thickness, LTa) indicates the distance from the anterior lens surface to the lens equatorial plane. **B** Illustrates a schematic of the lens structure in the 90°–270° section. In 2D results, Front R is the RAL, Back R is the RPL, and LE-Dia is the LD measured by the device. In 3D results, Front R and Back R represent the average RAL and RPL, LE-Dia represents the average LD from 3D device measurements. LTa, measured manually when the visual axis coincides with the lens axis, is 1.133 mm in **B**

Anterior lens surface curvature radius (RAL): The average of the steep-axis and flat-axis curvature values of the anterior lens surface in 3D mode, with specific values displayed in the "3D Result Front R" section of Fig. 1B. RAL reflects anterior lens surface curvature radius (i.e., anterior lens surface convexity): a smaller RAL indicates a more convex anterior lens surface.

Posterior lens surface curvature radius (RPL): The average of the steep-axis and flat-axis curvature values of the posterior lens surface in 3D mode, with specific values displayed in the "3D Result Back R" section of Fig. 1B.

Lens equatorial diameter (LD): The average of the longest and shortest distances between the intersection points of the anterior and posterior lens surfaces in 3D mode, with specific values displayed in the "3D Result LE-Dia" section of Fig. 1B.

#### Parameters automatically tracked and quantified by the CASIA 2 device (2D mode)

Coronal equatorial diameter (LDx): The LD value obtained from a two-dimensional cross-sectional scan in the coronal plane (0°–180°), defined as the distance between the two intersection points of the RAL and the RPL within this plane, which was subsequently used for the calculation of LV.

Sagittal equatorial diameter (LDy): The LD value obtained from a two-dimensional cross-sectional scan in the sagittal plane (90°–270°), defined as the distance between the two intersection points of the RAL and the RPL within this plane, which was subsequently used for the calculation of LV.

#### Manually measured parameters using the CASIA 2 device

Anterior lens thickness (LTa): In the scanning results display interface, when the visual axis coincided with the lens axis, the CASIA 2 manual measurement mode was used to evaluate the distances between the axis and the intersection points with the anterior lens surface and the lens equatorial plane at this angle. The specific value is displayed as 1.133 mm in Figure 1B, where the blue and yellow dashed lines represent the visual axis and lens axis.

### Calculation of lens volume (LV)

The lens was geometrically modeled as a regular oblate spheroid [7]. Its volume was calculated using the standard ellipsoid equation (Eq. 1). Anatomically, the lens can be partitioned into two hemi-ellipsoids of unequal thickness by the equatorial plane. The total LV was derived by summing the volumes of these two hemi-ellipsoids (superior and inferior to the equatorial plane) [5], as mathematically expressed in Eq. 2.

1 $$\:V\:=\:\frac{4}{3}\times\:\pi\:\times\:a\times\:b\times\:c$$

Here, a, b, and c represent the semi-axes of the ellipsoid along the three principal axes.

After incorporating the lens parameters, the equation becomes: a = LT/2, b = LD_x_/2, c = LD_y_/2.

2 $$\:LV\:=\:\frac{\pi\:}{6}\times\:LT\times\:\mathrm{L}\mathrm{D}\mathrm{x}\times\:\mathrm{L}\mathrm{D}\mathrm{y}$$

### Measurement of biometry

Ocular biometric parameters, including axial length (AL), lens thickness (LT), anterior chamber depth (ACD), mean keratometry (K_m_), and horizontal corneal diameter (white-to-white, WTW), were measured using the IOL Master 700 (Carl Zeiss Meditec, Jena, Germany). All examinations were performed by two experienced ophthalmologists following standardized protocols. Upon successful measurement, a green checkmark ‘√’ appeared on both the signal quality indicator and the analysis interface. The foveal morphology displayed on the macular analysis interface was carefully examined to verify proper patient fixation, after which the measurements were recorded.

### Measurement reliability

Because the LT_a_ parameter requires manual marking and measurement by operators on the imaging device, 50 patients were randomly selected from the entire study cohort, and repeated LT_a_ measurements were performed to evaluate measurement reliability. Two experienced operators (Operator A and Operator B), masked to each other’s results, independently measured LT_a_ twice using the CASIA 2 device, with a minimum interval of 5 days between sessions to minimize recall bias. The results are presented in Additional file 1.

### Statistical analyses

Statistical data analysis was performed using IBM SPSS Statistics for Windows, Version 25.0 (IBM Corp., Armonk, NY, USA). A *p* < 0.05 was considered statistically significant. All quantitative data were presented as mean ± standard deviation (SD). Differences in lens parameters between sexes were analyzed using independent-sample t-tests. One-way analysis of variance was employed to assess parameter differences among different age groups. Simple and multiple linear regression analyses were performed to evaluate the associations between lens parameters and age as well as other ocular biometric parameters. Generalized Additive Models (GAMs) were used to fit curves to nonlinearly distributed data points. Pearson correlation analysis was performed to evaluate the association between parameters, and correlation heatmaps and scatter plots were created using ChiPlot (https://www.chiplot.online/). Intraobserver and interobserver reliability were assessed using the intraclass correlation coefficient (ICC) under a two-way random-effects model with absolute agreement (ICC [1, 2]). Bland-Altman plots with 95% limits of agreement (LoA) were constructed to visually illustrate the agreement between repeated measurements. The 95% confidence intervals (CIs) for ICC estimates were derived using bootstrap resampling with 2000 iterations.

## Results

### Demographic data

A total of 356 cataract patients (356 eyes) were enrolled in this study. The cohort had a mean age of 66.48 ± 9.60 years (range: 40.00–89.00 years). Among them, 140 patients (39.33%) were male and 216 patients (60.67%) were female. Ocular biometric measurements yielded the following mean ± SD (range) values: AL, 23.92 ± 2.21 mm (range: 19.42–34.61 mm); K_m_, 44.25 ± 1.67D (range: 38.42–48.23 D); ACD, 3.05 ± 0.43 mm (range: 1.79–4.40 mm); WTW, 11.66 ± 0.42 mm (range: 10.50–12.80 mm).

### The distribution characteristics and correlations of three-dimensional lens parameters

Lens parameters (mean ± SD [range]) were as follows: RAL: 9.18 ± 1.32 mm (range: 6.04–14.10 mm); RPL: 5.52 ± 0.80 mm (range: 3.27–8.43 mm); LT: 4.49 ± 0.46 mm (range: 3.21–6.07 mm); LT_a_: 1.49 ± 0.34 mm (range: 0.65–2.54 mm); LD: 9.67 ± 0.95 mm (range: 6.51–12.25 mm); and LV: 224.71 ± 56.98 mm³ (range: 76.88–404.14 mm³).

Patients were stratified by RAL into three groups: low (RAL < 8.0 mm; *n* = 62, 17.4%), medium (8.0 mm ≤ RAL ≤ 10.5 mm; *n* = 236, 66.3%), and high (RAL > 10.5 mm; *n* = 58, 16.3%). Similarly, patients were stratified by LT into three groups: LT ≤ 4.0 mm (*n* = 50, 14.0%), 4.0 mm < LT < 5.0 mm (*n* = 259, 72.8%), and LT ≥ 5.0 mm (*n* = 47, 13.2%). By LD, patients were classified as short (LD < 8.5 mm; *n* = 55, 15.4%), medium (8.5 mm ≤ LD ≤ 10.5 mm; *n* = 243, 68.3%), and long (LD > 10.5 mm; *n* = 58, 16.3%). These stratifications were solely intended to describe the distribution of lens parameters across clinically relevant ranges.

**Fig. 2:**
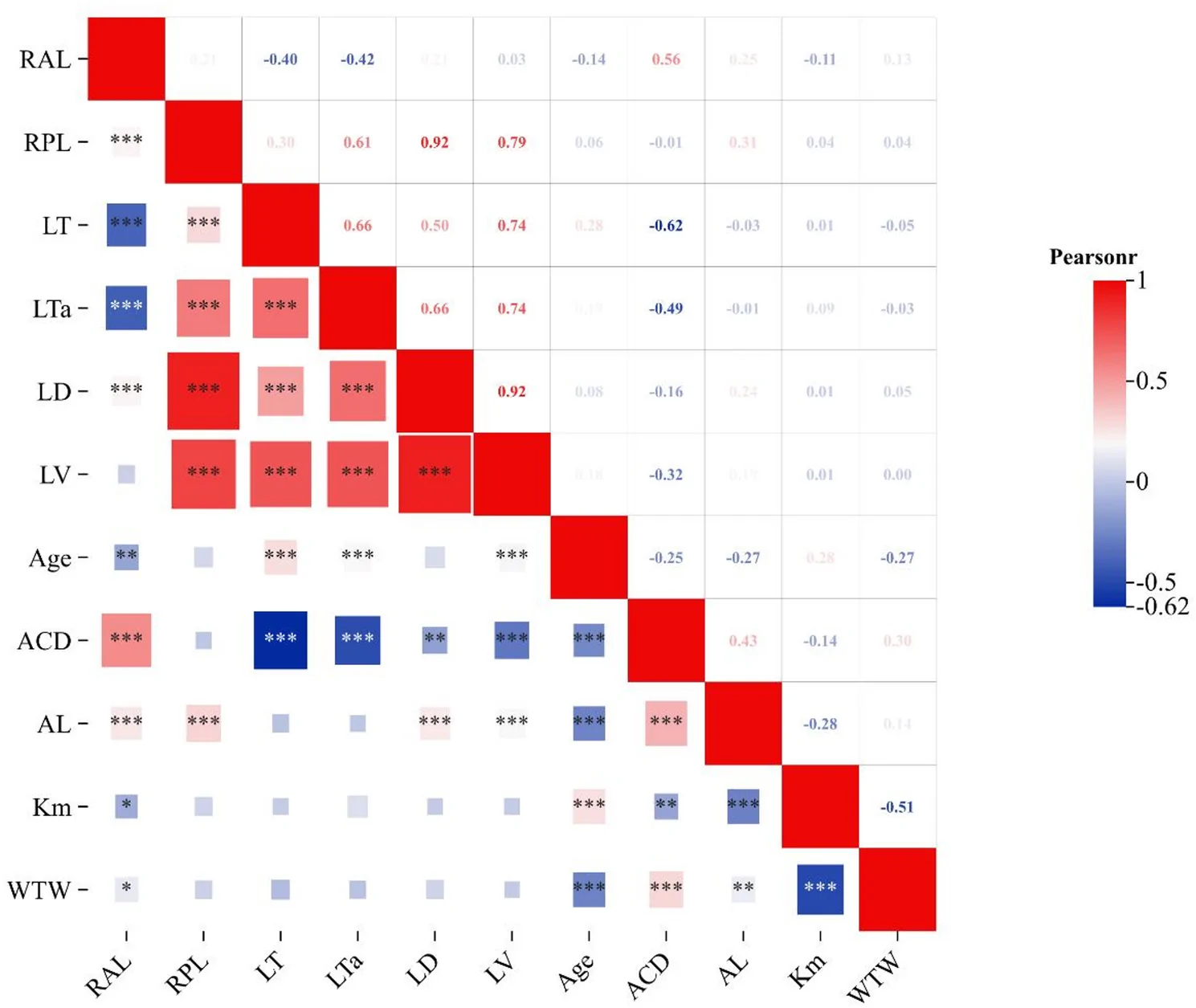
Pearson correlation analysis of all parameters. LV = lens volume; ACD = anterior chamber depth; AL = axial length; Km = mean keratometry; WTW = horizontal corneal diameter. Pearson correlation matrix of three−dimensional lens parameters (RAL, RPL, LT, LTa, LD, LV) and age and ocular biometric parameters (ACD, AL, Km, WTW) in 356 cataract patients. Each cell displays the Pearson correlation coefficient (r), with color gradient indicating direction and strength of correlation (light red to dark red: positive correlation; light blue to dark blue: negative correlation). Statistical significance: * *p* < 0.05; ** *p* < 0.01; *** *p* < 0.001.

Overall correlational analyses among all lens parameters across the entire cohort (*n* = 356) are presented in Fig. 2, with Pearson correlation coefficients revealing the following:

RAL was weakly positively correlated with RPL (*r* = 0.206, *p* < 0.001) and LD (*r* = 0.208, *p* < 0.001), moderately negatively correlated with LT (*r* = -0.396, *p* < 0.001) and LT_a_ (*r* = -0.418, *p* < 0.001), and showed no correlation with LV. Further analysis revealed that the LT ≤ 4.0 mm stratum had the largest mean RAL (9.82 ± 1.43 mm), whereas the LT ≥ 5.0 mm stratum demonstrated the smallest (8.23 ± 0.90 mm). The difference in RAL between these two strata was statistically significant (t = 6.516, *p* < 0.001).

RPL was weakly positively correlated with LT (*r* = 0.296, *p* < 0.001) and strongly positively correlated with LT_a_ (*r* = 0.612, *p* < 0.001), LD (*r* = 0.917, *p* < 0.001), and LV (*r* = 0.795, *p* < 0.001).

LT was moderately positively correlated with LD (*r* = 0.495, *p* < 0.001) and strongly positively correlated with LT_a_ (*r* = 0.655, *p* < 0.001) and LV (*r* = 0.741, *p* < 0.001).

LT_a_ was strongly positively correlated with LD (*r* = 0.660, *p* < 0.001) and LV (*r* = 0.742, *p* < 0.001). Similarly, LD was strongly positively correlated with LV (*r* = 0.921, *p* < 0.001).

### Comparison of lens parameters between different sexes

Table 1 presents the distribution characteristics of lens parameters stratified by sex. Statistical analysis demonstrated that none of the examined lens parameters showed statistically significant differences between sexes (*p* > 0.05).

**Table 1:** Statistical analysis of lens parameters between different sexes

Variables	Male (*n* = 140)	Female (*n* = 216)	*p*
RAL (mm)	9.261 ± 1.346	9.132 ± 1.295	0.367
RPL (mm)	5.525 ± 0.822	5.517 ± 0.786	0.930
LT (mm)	4.488 ± 0.485	4.486 ± 0.441	0.971
LT_a_ (mm)	1.494 ± 0.337	1.492 ± 0.340	0.960
LD (mm)	9.649 ± 0.951	9.676 ± 0.952	0.799
LV (mm^3^)	224.450 ± 60.025	224.884 ± 55.054	0.944

RAL = anterior lens surface curvature radius; RPL = posterior lens surface curvature radius; LT = lens thickness; LT_a_ = anterior lens thickness; LD = lens equatorial diameter; LV = lens volume; The p value represents the statistical analysis of differences in lens parameters between different sex groups (where *p* < 0.05 indicates a statistically significant difference)

### Comparison and analysis of lens parameters between different age groups

The data were stratified into five age groups: 40–50 years (Group 1), 51–60 years (Group 2), 61–70 years (Group 3), 71–80 years (Group 4), and over 80 years (Group 5). Tables 2 and 3 show that RAL showed statistically significant differences across age groups (*p* = 0.013). Post hoc analyses revealed that Group 4 exhibited statistically significant differences in RAL compared to Group 2 (*p* = 0.001) and Group 3 (*p* = 0.029), whereas no significant differences were observed between other group pairs (*p* > 0.05). Furthermore, RAL displayed a statistically significant negative linear correlation with age (*r* = -0.142, *p* = 0.007), decreasing at a rate of 0.019 mm per year of age increase.

LT demonstrated significant variations among age groups (*p* < 0.001). Post hoc tests indicated that Group 1 differed significantly from Groups 2–5 (all *p* < 0.05), and Group 2 showed significant differences when compared with Group 1 and Groups 3–5 (all *p* < 0.05). In contrast, no significant differences were observed among Groups 3, 4, and 5 (all *p* > 0.05). Notably, LT exhibited a significant positive linear correlation with age (*r* = 0.279, *p* < 0.001), with an annual increase rate of 0.013 mm.

LT_a_ demonstrated significant variation among age groups (*p* < 0.001). Post hoc analyses revealed statistically significant differences between Group 1 and Groups 3, 4, and 5 (*p* = 0.003, *p* = 0.001, and *p* = 0.043, respectively). A significant positive linear correlation was observed between LT_a_ and age (*r* = 0.194, *p* < 0.001), with an annual increase rate of 0.007 mm/year.

LV exhibited significant variations across age groups (*p* = 0.011). Post hoc analyses revealed statistically significant differences between Group 1 and Groups 2–5 (all *p* < 0.05), whereas no significant intergroup differences were observed among Groups 2–5 (all *p* > 0.05). A significant positive linear correlation was identified between LV and age (*r* = 0.177, *p* = 0.001), with an annual increase rate of 1.052 mm³/year. In contrast, neither RPL nor LD showed significant correlations with age (both *p* > 0.05).

After further stratification into ≤ 60 years and > 60 years groups, the correlations between lens parameters and age were analyzed separately. Subgroup analyses revealed that RAL and LT_a_ showed no statistically significant correlations with age in either subgroup (all *p* > 0.05). As presented in Table 3, LT demonstrated significant positive linear correlations with age in both subgroups (LT1: *r* = 0.246, *p* = 0.013; LT2: *r* = 0.147, *p* = 0.019), with annual increase rates of 0.022 mm/year for LT1 and 0.010 mm/year for LT2. For LV, significant positive correlation with age was observed in the ≤ 60 years subgroup (LV1: *r* = 0.206, *p* = 0.039), with an annual increase of 2.255 mm³/year. In contrast, in the > 60 years subgroup, LV was not statistically significantly correlated with age (*p* = 0.128).

**Table 2:** Analysis of differences in lens parameters between different age groups

Variables Mean ± SD	40–50 *N* = 18	51–60 *N* = 83	61–70 *N* = 127	71–80 *N* = 107	Age > 80 *N* = 21	*p*-value
RAL	9.393 ± 1.152	9.486 ± 1.167	9.215 ± 1.383	8.842 ± 1.258	9.350 ± 1.569	*p* = 0.013^*^
RPL	5.293 ± 0.866	5.534 ± 0.729	5.520 ± 0.747	5.496 ± 0.845	5.778 ± 1.054	*p* = 0.443
LT	4.098 ± 0.443	4.371 ± 0.428	4.509 ± 0.440	4.593 ± 0.468	4.606 ± 0.391	*p* < 0.001^*^
LT_a_	1.266 ± 0.222	1.437 ± 0.291	1.510 ± 0.326	1.540 ± 0.377	1.566 ± 0.380	*p* < 0.001^*^
LD	9.229 ± 0.833	9.674 ± 0.890	9.700 ± 0.893	9.644 ± 1.030	9.903 ± 1.140	*p* = 0.254
LV	184.161 ± 43.598	218.921 ± 53.512	228.264 ± 54.389	228.310 ± 59.226	242.569 ± 70.263	*p* = 0.011^*^

N = Number; The *P* value indicates the statistical difference analysis of lens parameters between different age groups. An asterisk (*) indicates a statistically significant difference (*p* < 0.05)

**Table 3:** Correlation and simple linear regression analysis of lens parameters with age

Variables	*r*	*p*_1_	β (95% CI)	*p*_2_
RAL	-0.142	0.007	-0.019 (-0.034 to -0.005)	0.007
LT	0.279	0.000	0.013 (0.009 to 0.018)	0.000
LT_1_	0.246	0.013	0.022 (0.005 to 0.040)	0.013
LT_2_	0.147	0.019	0.010 (0.002 to 0.019)	0.019
LT_a_	0.194	0.000	0.007 (0.003 to 0.010)	0.000
LV	0.177	0.001	1.052 (0.441 to 1.663)	0.001
LV_1_	0.206	0.039	2.255 (0.115 to 4.396)	0.039
LV_2_	0.096	0.128	0.857 (-0.248 to 1.962)	0.128

LT_1_ = linear relationship between lens thickness and age in patients ≤ 60 years old; LT_2_ = linear relationship between lens thickness and age in patients > 60 years old; LV_1_ = linear relationship between lens volume and age in patients ≤ 60 years old; LV_2_ = linear relationship between lens volume and age in patients > 60 years old; p_1_ = Pearson correlation analysis results; p_2_ = simple linear regression model results; β = slope value of the simulated univariate linear regression equation; 95% CI = 95% confidence interval

### Correlation analysis between three-dimensional lens parameters and biological parameters

**Fig. 3:**
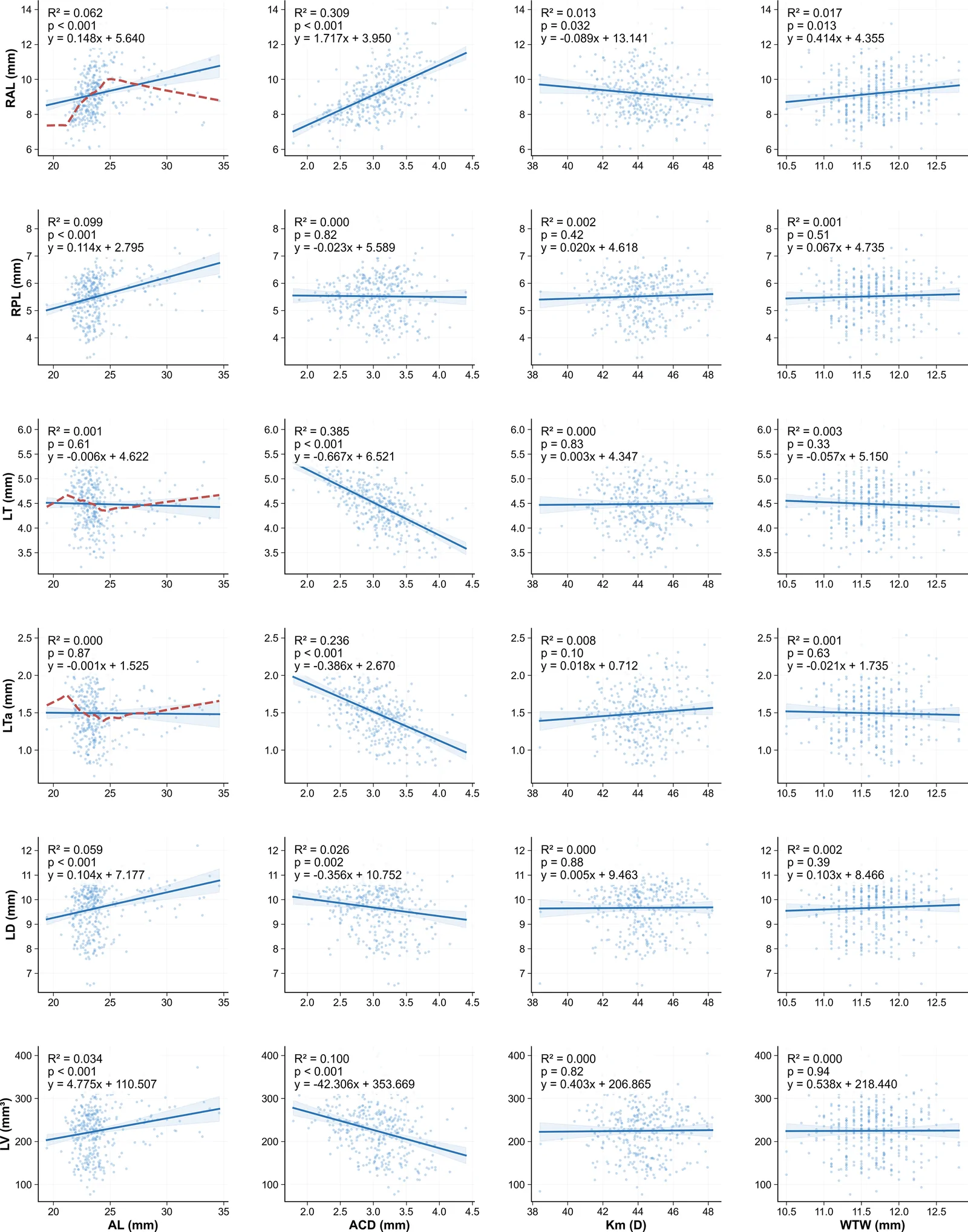
Regression analysis of lens parameters and ocular parameters. Scatter plots and regression analyses of three−dimensional lens parameters versus ocular biometric parameters. The grey shaded area indicates the range of the confidence interval. The blue solid line represents the linear regression analysis result between the two parameters. The optimal curves for RAL − AL, LT − AL, and LTa − AL were fitted using a generalized additive model (as indicated by the red dashed line), with breakpoints at AL = 24.54 mm, AL = 24.76 mm, and AL = 24.4 mm respectively.

As demonstrated in Figs. 2 and 3, the intergroup correlation analysis revealed the following:

RAL exhibited significant positive linear correlations with ACD (*r* = 0.556, *p* < 0.001) and WTW (*r* = 0.132, *p* = 0.013), and a significant negative linear correlation with K_m_ (*r* = -0.113, *p* = 0.032);

RPL demonstrated a significant positive linear correlation with AL (*r* = 0.315, *p* < 0.001) but showed no significant associations with other ocular parameters;

Both LT (*r* = -0.621, *p* < 0.001) and LT_a_ (*r* = -0.486, *p* < 0.001) showed significant negative linear correlations with RAL but were not significantly associated with other ocular parameters;

LD (*r* = -0.160, *p* = 0.002) and LV (*r* = -0.316, *p* < 0.001) exhibited significant negative correlations with RAL and significant positive linear correlations with AL (LD: *r* = 0.242, *p* < 0.001; LV: *r* = 0.185, *p* < 0.001), with no significant associations with other lens parameters (all *p* > 0.05).

Overall analysis showed that RAL was significantly positively correlated with AL (*r* = 0.249, *p* < 0.001), whereas LT and LT_a_ showed no significant linear correlations with AL (both *p* > 0.05). However, generalized additive model analyses revealed nonlinear relationships: RAL initially increased and subsequently decreased with increasing AL, while LT and LT_a_ demonstrated an initial decrease followed by an increase. As shown in Fig. 3, the analysis of nonlinear relationships estimated the inflection point of AL to be approximately 25.0 mm. AL subgroups were then categorized as AL < 25.0 mm and AL ≥ 25.0 mm to analyze correlations between AL and RAL, LT, and LT_a_ in different AL subgroups, with results presented in Table 4. For AL < 25.0 mm, LT (*r* = -0.120, *p* = 0.038) and LT_a_ (*r* = -0.165, *p* = 0.004) showed negative correlations with AL, while RAL (*r* = 0.400, *p* < 0.001) demonstrated a positive correlation with AL. For AL ≥ 25.0 mm, the correlation coefficients of LT, LT_a_, and RAL with AL showed opposite directions compared with the AL < 25.0 mm group.

**Table 4:** Analysis of correlations between lens parameters and AL in different AL subgroups

Variables	AL < 25.0 mm (*N* = 299 cases)		AL ≥ 25.0 mm (*N* = 57 cases)	
	*r* value	*p* value	*r* value	*p* value
RAL	0.400	0.000	-0.234	0.080
LT	-0.120	0.038	0.248	0.063
LT_a_	-0.165	0.004	0.205	0.125

RAL = anterior lens surface curvature radius; LT = lens thickness༛LT_a_ = anterior lens thickness

### Construction of multiple linear regression prediction equations for different lens parameters

Simple and multiple linear regression analyses were performed with age and standard ocular biometric parameters as independent variables to assess the associations between lens parameters and age as well as other ocular biometric parameters. Additionally, multiple linear regression predictive equations were established for each lens parameter. The detailed results are presented in Additional file 2 and Table 5.

Specifically, ACD explained 31.9% of the variance in RAL; AL, LT, and K_m_ explained 22.0% of the variance in RPL; LT, ACD, K_m_, AL, and WTW explained 46.3% of the variance in LT_a_; LT, AL, and K_m_ explained 32.5% of the variance in LD; and LT, AL, and ACD explained 60.3% of the variance in LV.

**Table 5:** Univariate and multivariate linear regression analysis of ocular biometric parameters and lens parameters

Dependent variable	Univariate			Multivariate		
	Predictor	β	*p*	Predictor	β	*p*
RAL	Age	-0.1416	0.007^**^	ACD	0.4996	< 0.001^***^
	AL	0.2489	< 0.001^***^			
	K_m_	-0.1135	0.0323^*^			
	ACD	0.5563	< 0.001^***^			
	WTW	0.1321	0.0126^*^			
	LT	-0.3957	< 0.001^***^			
RPL	AL	0.3148	< 0.001^***^	AL	0.3533	< 0.001^***^
	LT	0.2956	< 0.001^***^	K_m_	0.1735	0.003^**^
	-	-	-	LT	0.3221	< 0.001^***^
LT_a_	Age	0.1944	< 0.001^***^	AL	0.1386	0.005^**^
	ACD	-0.4861	< 0.001^***^	K_m_	0.1459	0.002^**^
	LT	0.6551	< 0.001^***^	ACD	-0.2464	< 0.001^***^
	-	-	-	WTW	0.1363	0.006^**^
	-	-	-	LT	0.5042	< 0.001^***^
LD	AL	0.2419	< 0.001^***^	AL	0.2513	< 0.001^***^
	ACD	-0.1598	0.002^**^	K_m_	0.1210	0.0242^*^
	LT	0.4955	< 0.001^***^	LT	0.5449	< 0.001^***^
LV	Age	0.1772	< 0.001^***^	AL	0.1769	< 0.001^***^
	AL	0.1852	< 0.001^***^	ACD	0.1214	0.0262^*^
	ACD	-0.3163	< 0.001^***^	LT	0.8176	< 0.001^***^
	LT	0.7406	< 0.001^***^	-	-	-

Note: Only predictors with *p* < 0.05 in the corresponding model are listed. β, standardized regression coefficient; adj.R², adjusted R². * *p* < 0.05; ** *p* < 0.01; *** *p* < 0.001. *n* = 356

## Discussion

Three-dimensional lens structural parameters are critically associated with ocular accommodative capacity, cataract progression, surgical design, IOL selection, and glaucoma-related pathogenesis. This study utilized CASIA 2 for three-dimensional lens parameter assessment [8] and IOLMaster 700 for standardized biometrics [9], enabling comprehensive investigation of their interrelationships.

RAL and RPL not only reflect the ciliary muscle accommodation ability in lens refractive power but also indicate the lens elasticity, zonular function, and relaxation status [10]. Pei et al. [11] identified RAL as a risk factor for zonular relaxation assessment; a smaller RAL often indicates zonular laxity, which has important implications for cataract surgical procedures. In our study, the RAL was 9.18 ± 1.32 mm, and RAL < 8.0 mm accounted for 17.4% (62 cases). Additionally, among the included patients, the RPL was 5.52 ± 0.80 mm. These mean values are lower than those reported in previous two-dimensional studies [12], but consistent with the three-dimensional results reported by Chen et al. [5] for cataract patients. This discrepancy occurs because the lens is an irregular ellipsoidal structure, and anterior and posterior surface curvature radii vary across different imaging planes. In our measurements, RAL and RPL values were typically larger in coronal Sect. (0°-180°). Furthermore, systematic errors may be introduced during image processing, including stretching, rotation, and distortion correction [13]. 

LT plays a critical role in IOL power and size determination [14]. In this study, the mean ± SD of LT was 4.49 ± 0.46 mm, consistent with those of studies using the IOLMaster 700 [15]. Miao et al. [16] identified thicker lenses (LT ≥ 5.0 mm) and anterior lens position as independent risk factors for primary angle-closure glaucoma. In our study population, 13.2% (47 cases) exhibited LT ≥ 5.0 mm, with this subgroup demonstrating the lowest mean ± SD of RAL (8.23 ± 0.91 mm) and ACD (2.60 ± 0.36 mm). These anatomical characteristics increase angle-closure glaucoma risk, supporting consideration of early cataract extraction in selected cases [17]. 

Modern IOL power formulas (e.g., Barrett Universal II, Holladay 2) rely on predicted ELP. Waring et al. demonstrated a strong correlation between LT_a_ and postoperative ELP measured by SS-OCT (*r* = 0.69, *p* < 0.01), suggesting that LT_a_ may improve ELP prediction and optimize IOL power calculations. [18] In this study, LT_a_ was 1.49 ± 0.34 mm, comparable to the previously reported 1.51 ± 0.28 mm [19]. 

LD reflects the capsular bag diameter, while LV reflects lens volume and serves as a proxy for estimating capsular bag volume. These parameters guide capsular tension ring selection, IOL model choice, and assessment of Toric IOL rotation risk. However, because the iris obscures the lens equator, anterior segment imaging devices such as the CASIA 2 SS-OCT cannot directly measure lens volume; mathematical modeling based on measurable linear parameters remains the standard approach. In this study, LD was 9.67 ± 0.95 mm and LV was 224.71 ± 56.98 mm³, consistent with Chen et al. [5] Minor discrepancies are likely attributable to differences in patient demographics and sample sizes. Martinez-Enriquez et al. [20] used a parametric 3D surface-reconstruction model and reported a mean lens volume of 205.5 mm³, within the range of our ellipsoid-based estimates; their validation against ex vivo measurements showed 96% accuracy (mean error: 9.30 ± 7.49 mm³). Appropriate ring selection based on capsular bag diameter can improve IOL centration and stability, reduce posterior capsule opacification, and enhance surgical outcomes [21, 22]. 

RAL showed significant negative correlations with both LT and LT_a_, while RPL showed positive correlations with these parameters, indicating that a thicker lens has a more convex anterior surface (smaller RAL) and a flatter posterior surface (larger RPL)—consistent with Satou et al. [23] RPL was also strongly positively correlated with LD and LV, suggesting that a larger posterior curvature radius is associated with greater lens diameter and volume. These correlations suggest that RPL is a useful indicator for lens morphology assessment, IOL design, and capsular tension ring selection.

Our study found no significant differences in lens parameters by sex, suggesting that lens morphology does not differ between males and females. Hashemi et al. [24] reported inconclusive findings regarding LT and sex, while most studies report slightly greater LT in males than females—potentially attributable to the generally longer AL in males [15]. In contrast, He et al. [25] found a tendency toward larger LT in females. These inconsistencies may reflect variations in demographic characteristics and sample sizes across studies.

Our study found a significant negative correlation between RAL and age (− 0.019 mm/year), reflecting age-related anterior lens structural changes, whereas RPL showed no significant age-related correlation. Li et al. [11] confirmed the RAL-age correlation, attributing it to age-related loss of anterior surface accommodation, and reported a positive LD-age correlation; however, we found no significant LD-age relationship, consistent with Richdale et al. [26] These inconsistencies likely reflect varying age distributions across study populations.

LT increased significantly with age (β = 0.013 mm/year, *p* < 0.001), consistent with Hashemi et al. [24] (0.018 mm/year) but lower than rates reported by Gupta et al. [27] (0.023 mm/year) and Wang et al. [28] (0.027 mm/year), and higher than the 0.003 mm/year reported by Jonas et al. [29] in a 9,046-eye model. These variations likely reflect differences in age distributions, ethnic backgrounds, sample sizes, and measurement methodologies. Similarly, LT_a_ increased significantly with age (β = 0.007 mm/year, *p* < 0.001), slightly exceeding the 0.005 mm/year and 0.004 mm/year reported in European and Indian populations, respectively, by Mohamed et al. [30] Multiple studies have established LTa as a critical parameter for optimizing next-generation IOL formulas, given its significant influence on postoperative IOL position and refractive shift [14]. Although the annual changes in RAL (− 0.019 mm/year) and LT (+ 0.013 mm/year) appear modest, their cumulative effects are clinically meaningful. Over 20 years from age 50 to 70, the projected 0.38 mm RAL decline (~ 4.1% of the cohort mean of 9.18 mm) progressively shifts patients toward the RAL < 8.0 mm threshold—observed in 17.4% of our cohort—below which zonular laxity and intraoperative capsular instability are significantly more likely, often necessitating capsular tension ring placement. Similarly, the cumulative 0.26 mm LT increase pushes more eyes toward LT ≥ 5.0 mm (already present in 13.2%), a well-established risk factor for angle-closure glaucoma due to anterior chamber shallowing and iris-lens diaphragm forward displacemen [31]. Moreover, increasing LT directly influences ELP, a critical determinant of IOL power calculation accuracy; incorporating LT improves the predictive performance of modern IOL formulas [32], and thicker lenses tend to induce a hyperopic shift in refractive outcomes when formulas do not account for this parameter [33]. 

LV also increased significantly with age (1.052 mm³/year). Lens swelling is the primary mechanism underlying both early cataract formation and age-related volume expansion [34]. Although lens parameters show overall linear age-related trends, the rates differ by age group. As shown in Table 3, patients aged ≤ 60 years demonstrated stronger correlations of LT and LV with age and higher annual growth rates, whereas in patients > 60 years, LT thickening plateaued and LV showed no significant age-related change—likely because the age-related RAL decrease offsets the minimal LT increase, resulting in no net volumetric change.

Our study showed a significant positive correlation between RAL and ACD, whereas LT, LT_a_, LD, and LV showed significant negative correlations with ACD. Additionally, RAL decreased markedly as AL decreased, indicating that shorter eyes have a more convex anterior lens surface—a finding consistent with the lens structural alterations characteristic of angle-closure glaucoma [35]. Previous studies have similarly reported an inverse relationship between LT and ACD [19, 36]. Our study also identified nonlinear RAL-AL, LT-AL, and LT_a_-AL relationships. In the AL < 25.0 mm range, RAL was positively correlated with AL, while LT and LT_a_ were negatively correlated with AL; above this threshold, RAL decreased (*r* = − 0.234) while LT (*r* = 0.248) and LT_a_ (*r* = 0.205) increased with further axial elongation, though these trends did not reach statistical significance, likely due to limited statistical power from the small sample size (57 eyes). This may reflect zonular overstretching in highly elongated eyes, reducing the ability to maintain lens shape and causing anterior surface protrusion; however, this hypothesis requires validation in larger samples [37]. Waring et al. [18] reported a weak negative correlation between AL and LT (*R* = -0.137). In contrast, Feng et al., in a large-scale investigation of 59,726 cataract patients, revealed a significant nonlinear relationship: LT demonstrated an inverse correlation with AL when AL < 27 mm, but transitioned to a positive correlation when AL exceeded this threshold. Importantly, they cautioned that conventional LT-AL correlation analyses may oversimplify the complex anatomical relationships between anterior segment structures/vitreous cavity depth and AL in pathological myopia, where ocular components exhibit disproportionate growth patterns [38]. Beyond RAL, LT, and LT_a_, LD and LV showed weak positive correlations with AL. Km showed a weak negative correlation with RAL, whereas WTW showed a weak positive correlation with RAL but no significant associations with other lens parameters.

Simple and multiple linear regression analyses were performed to evaluate the associations between lens parameters and age and other ocular biometric measurements. In the multiple regression models, ACD was the sole independent predictor of RAL, and LT was the strongest independent predictor of lens parameters. A noteworthy finding was that several variables exhibited shifts in significance between univariate and multiple regression analyses. Age, which showed significant univariate associations with RAL, LT_a_, and LV, lost its significance in the multiple regression models, suggesting that its effects on lens parameters were largely mediated through other ocular biometric factors. Conversely, Km emerged as a significant independent predictor of RPL, LT_a_, and LD in the multiple regression models despite lacking univariate significance for these parameters, indicating a suppressor effect when other variables were controlled. The established regression equations may facilitate objective estimation of lens parameters in clinical settings without specialized imaging equipment, thereby supporting personalized surgical planning and IOL selection.

This study has several limitations. First, the analysis was restricted to 356 patients with age-related cataract, which constitutes a relatively small sample size that may not fully represent the broader cataract population. In particular, the youngest age group (40–50 years) comprised only 18 patients, which limits the statistical power for detecting age-related differences in this subgroup and may render post hoc comparisons involving this group less reliable. Additionally, the AL ≥ 25.0 mm subgroup comprised only 57 eyes, limiting the statistical power to detect associations between lens parameters and axial length in this subgroup. Second, postoperative outcome data were not collected, precluding evaluation of the value of these parameters for predicting ELP. Third, although axial length was analyzed as a continuous variable, refractive error was not independently assessed as a confounder, and the absence of manifest refraction data limits the comprehensive characterization of refractive status. Fourth, cataract severity was not systematically graded or included as a covariate. Fifth, diabetes mellitus and glaucoma status were neither collected nor controlled for, despite both conditions being known to alter lens morphology. Finally, potential sources of bias in the volume estimates derived from the mathematical model, relative to the true lens volume, include the following: (1) the rotational symmetry assumption — the ellipsoid model presumes a circular equatorial cross-section, whereas the crystalline lens has distinct anterior and posterior surface curvatures, which may introduce volume estimation error; (2) iris obstruction precludes direct in vivo measurement of the equatorial diameter, necessitating extrapolation via a parametric model, and the simple intersection approach may yield inaccurate volume estimates; and (3) failure to correct for optical distortion may also introduce error in volume estimation.

Notwithstanding these limitations, the present study has several notable strengths. First, to our knowledge, this study comprehensively characterized three-dimensional lens parameters in patients with age-related cataract using SS-AS-OCT, thereby providing a more complete morphometric profile than conventional two-dimensional assessments. Second, rigorous measurement reliability for the manually assessed parameter (LT_a_) was established through both intraclass correlation coefficient and Bland-Altman analyses, confirming high intraobserver and interobserver agreement. Third, generalized additive model analyses identified nonlinear relationships between lens parameters and AL, revealing inflection points at approximately 25 mm AL; these findings offer novel insights into lens biometry in eyes with pathological myopia that are not captured by linear models alone. Fourth, predictive regression equations for lens parameters were established using routinely available ocular biometrics, which may facilitate clinical estimation of internal lens structural parameters without requiring specialized imaging equipment. Finally, the characterized parameters have direct clinical relevance: RAL informs zonular integrity assessment and capsular tension ring selection, LT_a_ may improve ELP prediction and IOL power calculation, and LT aids in angle-closure glaucoma risk stratification—collectively supporting personalized cataract surgical planning.

In conclusion, this study demonstrates that in cataract patients aged ≥ 40 years, anterior lens surface convexity, LT, and LV increased significantly with age, with more rapid progression rates before age 60 than thereafter. Multivariable analysis revealed that smaller ACD was associated with more pronounced anterior lens surface convexity and greater lens thickness. LT, LT_a_, and RAL showed nonlinear relationships with AL, where RAL was maximal and LT was thinnest at approximately 25 mm AL.

## Supplementary Information

Below is the link to the electronic supplementary material.

Supplementary Material 1

Supplementary Material 2

## Data Availability

The datasets generated and analysed during the current study are not publicly available due the data involve patients privacy but are available from the corresponding author on reasonable request.

## Abbreviations and acronyms

SS-AS-OCT: Swept-source anterior segment optical coherence tomography
RAL: Anterior lens surface curvature radius
RPL: Posterior lens surface curvature radius
LT: Lens thickness
LT_a_: Anterior lens thickness
LD: Lens diameter
LV: Lens volume
IOL: Intraocular lens
LD_x_: Coronal equatorial diameter
LD_y_: Sagittal equatorial diameter
AL: Axial length
ACD: Anterior chamber depth
K_m_: Mean keratometry
WTW: Horizontal corneal diameter (white-to-white)
GAMs: Generalized additive models
ELP: Effective lens position
