# Supplementary material for: Distribution characteristics and influencing factors of three-dimensional lens parameters in patients with age-related cataracts
Source: BMC Ophthalmol. 2026 Jun 29;26:572. doi: 10.1186/s12886-026-05062-7 (PMC13576091; doi:10.1186/s12886-026-05062-7)
Supplement: Supplementary file 1 [file 12886_2026_5062_MOESM1_ESM.docx]

Intraobserver ICC (2,1) values were 0.9992 (95% CI: 0.9986–0.9995) for Operator A and 0.9987 (95% CI: 0.9979–0.9991) for Operator B. Interobserver ICC (2,1) values were 0.9977 (95% CI: 0.9962–0.9986) at Session 1 and 0.9980 (95% CI: 0.9968–0.9987) at Session 2. All ICC values exceeded 0.99. Bland-Altman analysis demonstrated negligible mean differences and narrow 95% limits of agreement, with no systematic bias observed across the measurement range. These findings demonstrate high reliability and reproducibility of the two operators in parameter measurement.

### Table 1. Descriptive Statistics of LT_a_ Measurements

| **Measurement** | **Mean ± SD (mm)** | **Min (mm)** | **Max (mm)** | **Range (mm)** |
| --- | --- | --- | --- | --- |
| Operator A – Session 1 | 1.611 ± 0.370 | 0.943 | 2.338 | 1.395 |
| Operator A – Session 2 | 1.612 ± 0.368 | 0.932 | 2.338 | 1.406 |
| Operator B – Session 1 | 1.600 ± 0.366 | 0.920 | 2.350 | 1.430 |
| Operator B – Session 2 | 1.598 ± 0.367 | 0.934 | 2.313 | 1.379 |

### Table 2. Intraclass Correlation Coefficients for LT_a_ Measurements

| **Comparison** | **ICC(2,1)** | **95% CI** |
| --- | --- | --- |
| Intraobserver – Operator A | 0.9992 | [0.9986, 0.9995] |
| Intraobserver – Operator B | 0.9987 | [0.9979, 0.9991] |
| Interobserver – Session 1 | 0.9977 | [0.9962, 0.9986] |
| Interobserver – Session 2 | 0.9980 | [0.9968, 0.9987] |

### Table 3. Bland-Altman Analysis for LT_a_ Measurements

| **Comparison** | **Mean Difference (mm)** | **95% LoA Lower (mm)** | **95% LoA Upper (mm)** |
| --- | --- | --- | --- |
| Intraobserver – Operator A | -0.0016 | -0.0315 | 0.0283 |
| Intraobserver – Operator B | 0.0021 | -0.0351 | 0.0392 |
| Interobserver | 0.0106 | -0.0341 | 0.0553 |

### Figure 1. Bland-Altman Plots for LT_a_ Measurement Agreement

(A) Intraobserver agreement – Operator A

(B) Intraobserver agreement – Operator B

(C) Interobserver agreement
