# Supplementary material for: Distribution characteristics and influencing factors of three-dimensional lens parameters in patients with age-related cataracts
Source: BMC Ophthalmol. 2026 Jun 29;26:572. doi: 10.1186/s12886-026-05062-7 (PMC13576091; doi:10.1186/s12886-026-05062-7)
Supplement: Supplementary file 2 [file 12886_2026_5062_MOESM2_ESM.docx]

Supplementary Table S1

Univariate and multivariate linear regression analysis for ocular biometric parameters

|  |  | **Univariate** |  |  |  |  | **Multivariate** |  |  |  |
| --- | --- | --- | --- | --- | --- | --- | --- | --- | --- | --- |
| **Dependent variable** | **Predictor** | **r** | **R²** | **adj.R²** | **β** | **p** | **R²** | **adj.R²** | **β** | **p** |
| **RAL** | Age | -0.1416 | 0.0201 | 0.0173 | -0.1416 | 0.007^**^ | **0.3187** | **0.3070** | 0.0213 | 0.6716 |
|  | AL | 0.2489 | 0.0620 | 0.0593 | 0.2489 | <0.001^***^ |  |  | 0.0273 | 0.6251 |
|  | Km | -0.1135 | 0.0129 | 0.0101 | -0.1135 | 0.0323^*^ |  |  | -0.0673 | 0.2107 |
|  | ACD | 0.5563 | 0.3095 | 0.3075 | 0.5563 | <0.001^***^ |  |  | 0.4996 | <0.001^***^ |
|  | WTW | 0.1321 | 0.0175 | 0.0147 | 0.1321 | 0.0126^*^ |  |  | -0.0564 | 0.3128 |
|  | LT | -0.3957 | 0.1566 | 0.1542 | -0.3957 | <0.001^***^ |  |  | -0.0930 | 0.1462 |
| **RPL** | Age | 0.0621 | 0.0039 | 0.0010 | 0.0621 | 0.2427 | **0.2199** | **0.2065** | 0.0565 | 0.2924 |
|  | AL | 0.3148 | 0.0991 | 0.0966 | 0.3148 | <0.001^***^ |  |  | 0.3533 | <0.001^***^ |
|  | Km | 0.0425 | 0.0018 | -0.0010 | 0.0425 | 0.4238 |  |  | 0.1735 | 0.003^**^ |
|  | ACD | -0.0121 | 0.0001 | -0.0027 | -0.0121 | 0.8198 |  |  | 0.0482 | 0.5279 |
|  | WTW | 0.0353 | 0.0012 | -0.0016 | 0.0353 | 0.5066 |  |  | 0.0902 | 0.1316 |
|  | LT | 0.2956 | 0.0874 | 0.0848 | 0.2956 | <0.001^***^ |  |  | 0.3221 | <0.001^***^ |
| **LT_a_** | Age | 0.1944 | 0.0378 | 0.0351 | 0.1944 | <0.001^***^ | **0.4631** | **0.4539** | 0.0257 | 0.5633 |
|  | AL | -0.0086 | 0.0001 | -0.0028 | -0.0086 | 0.8711 |  |  | 0.1386 | 0.005^**^ |
|  | Km | 0.0870 | 0.0076 | 0.0048 | 0.0870 | 0.1013 |  |  | 0.1459 | 0.002^**^ |
|  | ACD | -0.4861 | 0.2363 | 0.2341 | -0.4861 | <0.001^***^ |  |  | -0.2464 | <0.001^***^ |
|  | WTW | -0.0257 | 0.0007 | -0.0022 | -0.0257 | 0.6289 |  |  | 0.1363 | 0.006^**^ |
|  | LT | 0.6551 | 0.4292 | 0.4275 | 0.6551 | <0.001^***^ |  |  | 0.5042 | <0.001^***^ |
| **LD** | Age | 0.0787 | 0.0062 | 0.0034 | 0.0787 | 0.1384 | **0.3251** | **0.3135** | -0.0012 | 0.9811 |
|  | AL | 0.2419 | 0.0585 | 0.0559 | 0.2419 | <0.001^***^ |  |  | 0.2513 | <0.001^***^ |
|  | Km | 0.0080 | 0.0001 | -0.0028 | 0.0080 | 0.8801 |  |  | 0.1210 | 0.0242^*^ |
|  | ACD | -0.1598 | 0.0255 | 0.0228 | -0.1598 | 0.002^**^ |  |  | 0.0636 | 0.3702 |
|  | WTW | 0.0454 | 0.0021 | -0.0008 | 0.0454 | 0.3931 |  |  | 0.0794 | 0.1537 |
|  | LT | 0.4955 | 0.2455 | 0.2434 | 0.4955 | <0.001^***^ |  |  | 0.5449 | <0.001^***^ |
| **LV** | Age | 0.1772 | 0.0314 | 0.0287 | 0.1772 | <0.001^***^ | **0.6033** | **0.5964** | 0.0131 | 0.7330 |
|  | AL | 0.1852 | 0.0343 | 0.0316 | 0.1852 | <0.001^***^ |  |  | 0.1769 | <0.001^***^ |
|  | Km | 0.0118 | 0.0001 | -0.0027 | 0.0118 | 0.8243 |  |  | 0.0795 | 0.0531 |
|  | ACD | -0.3163 | 0.1000 | 0.0975 | -0.3163 | <0.001^***^ |  |  | 0.1214 | 0.0262^*^ |
|  | WTW | 0.0040 | 0.0000 | -0.0028 | 0.0040 | 0.9406 |  |  | 0.0282 | 0.5079 |
|  | LT | 0.7406 | 0.5485 | 0.5472 | 0.7406 | <0.001^***^ |  |  | 0.8176 | <0.001^***^ |

Note: β, standardized regression coefficient; adj.R², adjusted R²; r, Pearson correlation coefficient.

* p < 0.05; ** p < 0.01; *** p < 0.001.

Univariate: simple linear regression for each predictor separately. Multivariate: multiple linear regression with all predictors entered simultaneously (Enter method). n = 356.

Multivariate regression equations: RAL = 9.5074 + 0.0029 × Age + 0.0163 × AL − 0.0531 × Km + 1.5419 × ACD − 0.1767 × WTW − 0.2669 × LT; RPL = -6.3358 + 0.0047 × Age + 0.1279 × AL + 0.0832 × Km + 0.0904 × ACD + 0.1719 × WTW + 0.5622 × LT; LTa = -2.7438 + 0.0009 × Age + 0.0212 × AL + 0.0296 × Km − 0.1958 × ACD + 0.1100 × WTW + 0.3726 × LT; LD = -3.5641 − 0.0001 × Age + 0.1081 × AL + 0.0689 × Km + 0.1419 × ACD + 0.1798 × WTW + 1.1305 × LT; LV = -560.2296 + 0.0775 × Age + 4.5623 × AL + 2.7161 × Km + 16.2315 × ACD + 3.8317 × WTW + 101.7035 × LT.
